# Mapping the Interactions of Dengue Virus NS1 Protein with Human Liver Proteins Using a Yeast Two-Hybrid System: Identification of C1q as an Interacting Partner

**DOI:** 10.1371/journal.pone.0057514

**Published:** 2013-03-14

**Authors:** Emiliana M. Silva, Jonas N. Conde, Diego Allonso, Mauricio L. Nogueira, Ronaldo Mohana-Borges

**Affiliations:** 1 Laboratório de Genômica Estrutural, Instituto de Biofísica Carlos Chagas Filho, Universidade Federal do Rio de Janeiro, Rio de Janeiro, Rio de Janeiro, Brazil; 2 Departamento de Doenças Dermatológicas, Infecciosas e Parasitárias, Faculdade de Medicina de São José do Rio Preto, São José do Rio Preto, São Paulo, Brazil; Centro de Pesquisas René Rachou, Brazil

## Abstract

Dengue constitutes a global health concern. The clinical manifestation of this disease varies from mild febrile illness to severe hemorrhage and/or fatal hypovolemic shock. Flavivirus nonstructural protein 1 (NS1) is a secreted glycoprotein that is displayed on the surface of infected cells but is absent in viral particles. NS1 accumulates at high levels in the plasma of dengue virus (DENV)-infected patients, and previous reports highlight its involvement in immune evasion, dengue severity, liver dysfunction and pathogenesis. In the present study, we performed a yeast two-hybrid screen to search for DENV2 NS1-interacting partners using a human liver cDNA library. We identified fifty genes, including human complement component 1 (C1q), which was confirmed by coimmunoprecipitation, ELISA and immunofluorescence assays, revealing for the first time the direct binding of this protein to NS1. Furthermore, the majority of the identified genes encode proteins that are secreted into the plasma of patients, and most of these proteins are classified as acute-phase proteins (APPs), such as plasminogen, haptoglobin, hemopexin, α-2-HS-glycoprotein, retinol binding protein 4, transferrin, and C4. The results presented here confirm the direct interaction of DENV NS1 with a key protein of the complement system and suggest a role for this complement protein in the pathogenesis of DENV infection.

## Introduction

Dengue constitutes a major global health concern. It is estimated that nearly half of the worldwide population lives in risk areas and that fifty to one hundred million infections occur each year, including 500,000 hospitalizations of patients with severe dengue illness [1], [2]. Dengue virus (DENV) is a member of the *Flaviviridae* family, and it cocirculates as four distinct antigenic serotypes (DENV1–4). Infection with DENV may induce a spectrum of symptoms varying from none to severe plasma leakage, hemorrhage and organ impairment [3]. The mechanism underlying endothelial cell dysfunction and vascular leakage is of primary importance; however, it is far from being understood. Several studies have been published attempting to elucidate the principal phenomenon that leads to severe disease. Indeed, it has been established that the risk of developing severe dengue may be associated with secondary heterologous infection, leading to the phenomenon of antibody-dependent enhancement (ADE) [4], in addition to high viral loads [5]–[7] and multiple host factors including age, gender, genotype and prior immunity, among others [8], [9]. Disease severity can also be correlated to circulating levels of certain cytokines and chemokines such as tumor necrosis factor-alpha (TNF-α), interleukin 1β (IL-1β), interleukin 6 (IL-6), interleukin 10 (IL-10), interferon-gamma (IFN-γ), interleukin 8 (IL-8), macrophage inflammatory protein 1 (MIP-1) [10]–[18], and complement components (C3a, C5a, factor D and factor H) [19]–[22]. Despite the fact that several cell types and tissues have been described as potential sites for DENV replication and release of plasma immune mediators, the liver is one of the most important infection target organs [23], [24].

The flavivirus nonstructural protein 1 (NS1) is a 50 kDa intracellular homodimeric glycoprotein that plays a pivotal role in DENV replication [25], and there is evidence that it also plays an important role in dengue severity and pathogenesis [6], [26]. Although lacking a membrane-anchoring domain, the NS1 protein associates with organelle membranes and in particular with lipid-rafts, suggesting that it is involved in signal transduction pathways [27]. This association likely occurs via a GPI anchor [28]. The DENV NS1 protein is also secreted into the plasma as a lipid-associated barrel-shaped hexamer that is detectable in patient serum in the first few days after the onset of clinical symptoms in both primary and secondary infections [29], [30]. Recent reports highlight the involvement of the NS1 protein in the modulation of the complement system and the vascular leakage process, which facilitate immune complex formation [20]. Moreover, the NS1 protein elicits autoantibodies that react with platelets and extracellular matrix proteins [31] or that interfere with endothelial antibody-dependent, complement-mediated cytolysis [10]. DENV NS1 also exhibits complement antagonism by binding directly to complement proteins, including C4 and C1s, which leads to the degradation of C4 in solution and, consequently, to the inhibition of complement activation [32]. Alcon-LePoder and coworkers (2005) demonstrated that the liver is the major site for NS1 protein accumulation and preincubation of hepatocytes with soluble NS1 enhances subsequent infection by a homologous strain of DENV [33]. However, the mechanism by which NS1 is involved in dengue pathogenesis remains unclear.

To understand the role of the NS1 protein in DENV infection and pathogenesis, a yeast two-hybrid system was used to screen for the interacting partners of the DENV NS1 protein using a human liver cDNA library. We identified 50 different NS1-interacting partners, including the C1q protein. Coimmunoprecipitation, ligand binding ELISA, and immunofluorescence assays were also performed to confirm the direct binding of NS1 to human C1q. These results indicate the association of the DENV NS1 protein with another complement component, which suggests a role for this complement protein in DENV pathogenesis.

## Results

### Identification of DENV NS1-interacting partners using the yeast two-hybrid system

Considering the importance of the NS1 protein during DENV infection and its possible involvement with liver dysfunction [33], we aimed to understand its role in DENV infection and pathogenesis. Therefore, we performed a yeast two-hybrid screening to detect novel putative NS1 interacting-partners using a liver cDNA library. First, to determine whether the recombinant yeast AH109 cells expressed the NS1 protein, we performed a Western blot assay using the anti-NS1 polyclonal antibody (as described elsewhere [34]). We observed that the NS1 protein was properly expressed in these cells and did not interfere with cell growth (data not shown). Previous studies from our laboratory revealed that the NS1 protein expressed in yeast cells preserved its structural properties and was found as a glycosylated dimer (unpublished data).

**Figure 1:**
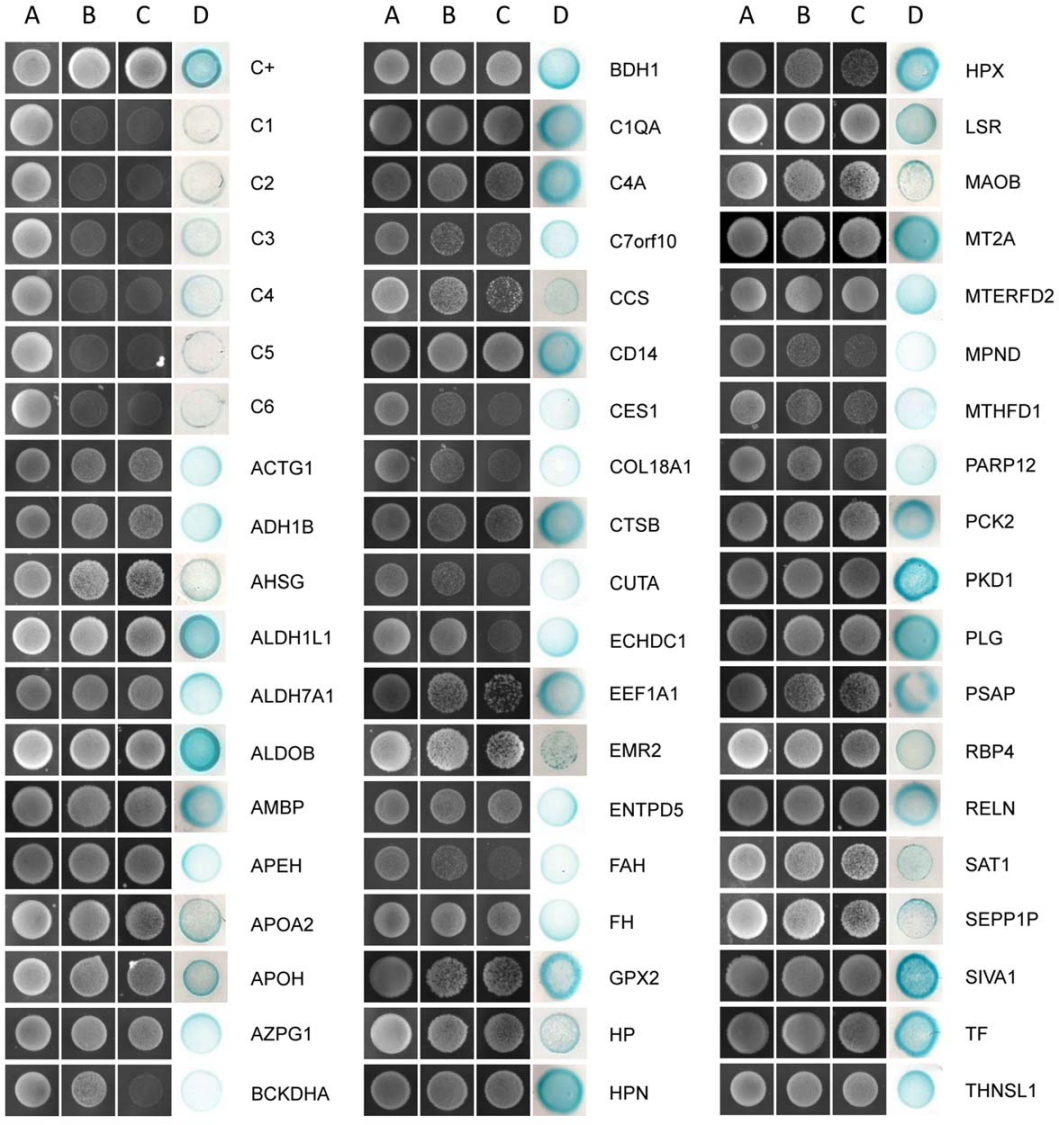
Plasmid linkage assays for transformants identified using DENV2 NS1 as bait in the yeast two-hybrid screening. Transformants containing the bait and prey plasmids were visualized by their growth on double drop-out media (SD–Leu–Trp; column A). Putative interacting partners were visualized by their growth on triple (SD–His–Leu–Trp; column B) and quadruple drop-out media (SD–Ade–His–Leu–Trp; column C) and by the blue color staining of the colony-lift filter assay (column D) indicating HIS3, ADE2 and *lacZ* reporter gene activation, respectively. AH109 yeast cells cotransformed with the plasmids pGBKT7-53 (murine p53 fused to the GAL4 DNA-binding domain) and pGADT7-T (SV40 large T-antigen fused to the GAL4 activation domain) served as positive controls (C+). AH109 cotransformed with the plasmids pGBKT7-NS1 and pGADT7-AD (C1), pGBKT7-NS1 and pGADT7-T (C2), pGBKT7 and pGADT7 (C3), pGBKT7 and pGADT7-T (C4), pGBKT7-Lam (laminin C) and pGADT7 (C5), and pGBKT7-Lam and pGADT7-T (C6) served as negative controls. The gene name for each acronym is detailed in Table 1.

Next, to select putative positive clones, we first grew the recombinant cells containing the bait and prey plasmids in triple drop-out media. This selection yielded 2,080 colonies. We identified the recombinant colonies containing a positive NS1 protein-interacting partner by HIS3, ADE2 and *lacZ* reporter gene activation, as visualized by cell growth on triple and quadruple drop-out media plates and by blue color staining following the colony-lift assays (Fig. 1). This analysis led to the identification of 50 different genes, including genes that encode the complement proteins C1q and C4 in addition to genes that encode several acute-phase proteins (APPs), such as plasminogen, haptoglobin, and hemopexin, among others (Table 1). Interestingly, the interaction of NS1 with the complement C4 protein has been described previously [32].

**Table 1:** Human liver proteins that interact with DENV2 NS1 protein identified by yeast two-hybrid screening.

Abbreviation	Gene name	NCBI ID	No. of clones detected
ACTG1	actin, gamma 1	NG_011433.1	2
ADH1B	alcohol dehydrogenase 1B (class I), beta polypeptide	NG_011435.1	4
AHSG	alpha-2-HS-glycoprotein	NG_011436.1	2
ALDH1L1	aldehyde dehydrogenase 1 family, member L1	NG_012260.1	2
ALDH7A1	aldehyde dehydrogenase 7 family, member A1	NG_008600.2	5
ALDOB	aldolase B, fructose-bisphosphate	NG_012387.1	13
AMBP	alpha-1-microglobulin/bikunin precursor	NM_001633.3	5
APEH	N-acylaminoacyl-peptide hydrolase	NM_001640.3	2
APOA2	apolipoprotein A-II	NM_001643.1	1
APOH	apolipoprotein H (beta-2-glycoprotein I)	NG_012045.1	5
AZGP1	alpha-2-glycoprotein 1, zinc-binding	NM_001185.3	1
BCKDHA	branched chain keto acid dehydrogenase E1, alpha polypeptide	NM_000709.3	1
BDH1	3-hydroxybutyrate dehydrogenase, type 1	NM_004051.4	1
C1QA	complement component 1, q subcomponent, A chain	NG_007282.1	2
C4A	complement component 4A	NM_007293.2	2
C7orf10	C7orf10 chromosome 7 open reading frame 10	NM_001193311.1	1
CCS	copper chaperone for superoxide dismutase	NM_005125.1	1
CD14	CD14 molecule	NG_023178.1	2
CES1	carboxylesterase 1	NG_012057.1	1
COL18A1	collagen, type XVIII, alpha 1	NM_030582.3	1
CTSB	cathepsin B	NG_009217.1	1
CUTA	cutA divalent cation tolerance homolog (E. coli)	NM_001014840.1	1
ECHDC1	enoyl CoA hydratase domain containing 1	NM_001002030.1	2
EEF1A1	eukaryotic translation elongation factor 1 alpha 1	NM_001402.5	3
EMR2	egf-like module containing, mucin-like, hormone receptor-like 2	NM_152916.1	1
ENTPD5	ectonucleoside triphosphate diphosphohydrolase 5	NM_001249.2	1
FAH	fumarylacetoacetate hydrolase (fumarylacetoacetase)	NG_012833.1	1
FH	fumarate hydratase	NG_012338.1	1
GPX2	glutathione peroxidase 2	NM_002083.2	1
HP	haptoglobin	NG_012651.1	1
HPN	hepsin	NM_002151.2	1
HPX	hemopexin	NM_000613.2	1
LSR	lipolysis stimulated lipoprotein receptor	NM_205834.2	1
MAOB	monoamine oxidase B	NG_008723.1	1
MPND	MPN domain containing	NM_001159846.1	1
MT2A	metallothionein 2A	NM_005953.3	13
MTERFD2	MTERF domain containing 2	NM_182501.3	1
MTHFD1	methylenetetrahydrofolate dehydrogenase (NADP+ dependent) 1, methenyltetrahydrofolate cyclohydrolase, formyltetrahydrofolate synthetase	NG_012450.1	1
PARP12	poly (ADP-ribose) polymerase family, member 12	NM_022750.2	2
PCK2	phosphoenolpyruvate carboxykinase 2 (mitochondrial)	NG_008162.1	2
PKD1	polycystic kidney disease 1	NG_008617.1	1
PLG	plasminogen	NG_016200.1	2
PSAP	prosaposin	NM_001042465.1	2
RBP4	retinol binding protein 4, plasma	NG_009104.1	2
RELN	reelin	NG_011877.1	4
SAT1	spermidine/spermine N1-acetyltransferase 1	NG_012929.1	1
SEPP1	selenoprotein P, plasma, 1	NM_001085486.1	1
SIVA1	SIVA1, apoptosis-inducing factor	NM_006427.3	7
TF	transferrin	NM_001063.3	1
THNSL1	threonine synthase-like 1	NM_024838.4	1

**Figure 2:**
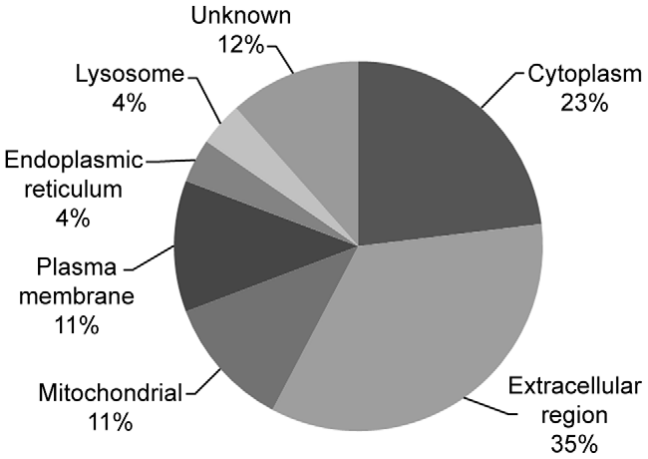
Cellular localization of DENV2 NS1 interacting-partners identified by yeast two-hybrid screening.

Thus, to identify the corresponding proteins of these identified genes, we subjected their sequences to BLASTX analysis (available at the NCBI website). Next, we arranged them according to their primary cellular localization, as shown in Figure 2. We determined that NS1-interacting partners belonged to a wide range of protein classes including extracellular milieu-released proteins (35% of all proteins screened by the yeast-two hybrid system) that includes, for example, the apolipoprotein A2 and H, C1q, C4, haptoglobin, hemopexin, plasminogen, and transferrin; cytoplasmic-resident proteins (23% of all proteins) such as alcohol dehydrogenase 1B, aldehyde dehydrogenase 1 and 7, and eukaryotic translation elongation factor 1; mitochondrial-resident proteins (11% of all proteins), such as, for example, 3-hydroxybutyrate dehydrogenase, monoamine oxidase B and phosphoenolpyruvate carboxykinase 2; plasma membrane proteins (11% of all proteins) such as CD14; endoplasmic reticulum-resident proteins (4% of all proteins) such as carboxylesterase 1; and lysosomal proteins (4% of all proteins) such as prosaposin. These results indicate the ability of the NS1 protein to interact with different classes of host proteins, primarily those localized in the extracellular region.

### DENV NS1 directly interacts with C1q

**Figure 3:**
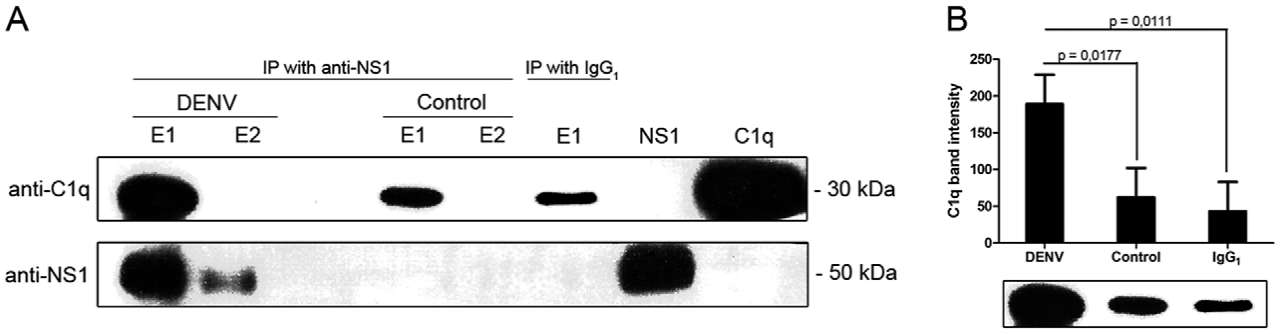
Coimmunoprecipitation of human C1q and NS1 proteins. (A) Purified NS1 from the supernatants of DENV-infected BHK cells and purified human C1q protein were immunoprecipitated with anti-NS1 polyclonal antibody, and the eluted fractions 1 and 2 (E1 and E2) were subjected to Western blot analysis. Bands of approximately 30 and 50 kDa corresponding to C1q and NS1, respectively, were observed in the elution fraction of the coimmunoprecipitation. (B) C1q was capable of binding anti-NS1 antibody, although the band intensity in the DENV lane E1 was more than four-fold intense than that in the control lane E1. The IgG_1_-coated resin eluted a similar amount of C1q as the control. Error bars indicate the standard deviation from three independent experiments, and the *p* value denotes significant differences from the control.

Because the interaction of flavivirus NS1 with complement system proteins and its regulators appears to play an important role in the immune evasion process [32], [35], [36], we focused our studies on the confirmation of the DENV NS1 and C1q interaction. First, we performed a coimmunoprecipitation assay in which the anti-NS1 polyclonal antibody was immobilized to the resin and subsequently incubated with NS1, which was purified in its hexameric form from the supernatants of DENV-infected BHK cells, and purified human C1q protein. Because C1q is able to bind the F_c_ region of antibodies [37], we also assessed the interaction of purified C1q with the anti-NS1-coated resin (control). The elution fractions were then analyzed by Western blot using another specific anti-NS1 polyclonal antibody [34] and an anti-C1q monoclonal antibody. Two bands of approximately 30 and 50 kDa corresponding to C1q and NS1, respectively, were obtained, indicating that C1q coimmunoprecipitated with the NS1 protein (Fig. 3A). As expected, a band of approximately 30 kDa was also observed in the control experiment (Fig. 3A). However, the band intensity for C1q in the presence of NS1 was more than four-fold higher than that in the absence of NS1 (control), and this difference is statistically significant (*p* = 0.0177, Fig. 3B). These findings confirm the interaction between the NS1 and C1q proteins as determined by the yeast two-hybrid screening.

Previous studies have shown that anti-NS1 antibodies are able to cross-react with some human proteins, such as blood clotting factors and adhesion molecules [31], [38]. Therefore, to verify whether the C1q precipitation in the control experiment was caused by its binding to the F_c_ region of the anti-NS1 antibody or its cross interaction, we performed an identical experiment as described above using a nonspecific IgG_1_-coated resin. We observed a sharp band of approximately 30 kDa in the elution fraction, indicating that the C1q protein was also immunoprecipitated by IgG_1_ at low levels (Fig. 3A). Analysis of the band intensity revealed that there is no significant difference between coimmunoprecipitation using IgG_1_ and the control experiments, but there is a significant difference between coimmunoprecipitation using IgG_1_ and coimmunoprecipitation in the presence of NS1 (*p* = 0.0111, Fig. 3B). These results indicate that the anti-NS1 antibody does not cross-react with C1q.

**Figure 4:**
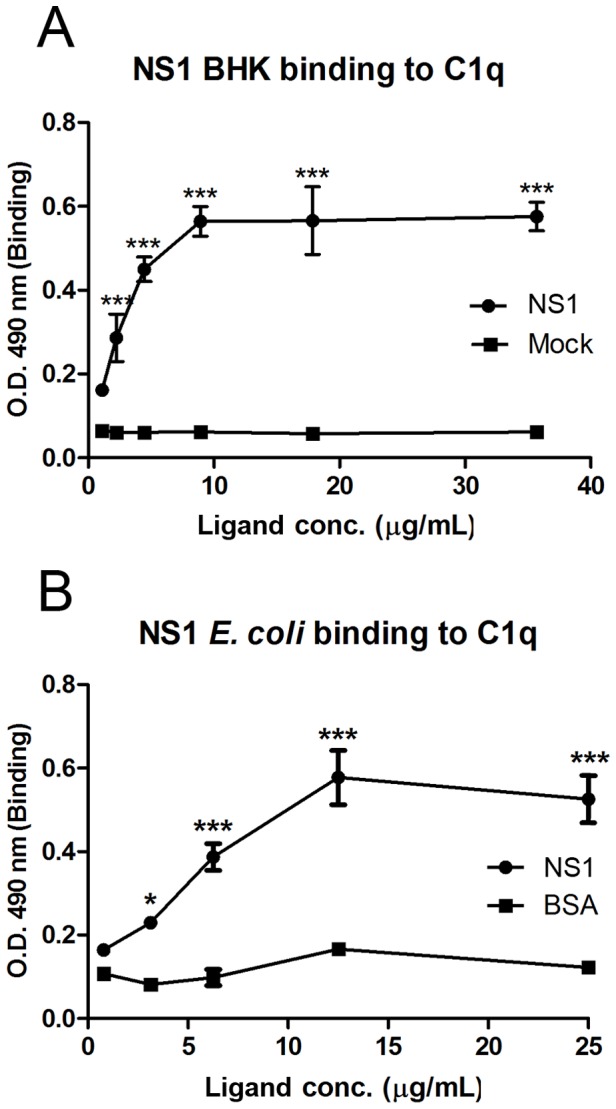
DENV NS1 directly binds human C1q in an ELISA assay. (A) Microtiter plates were coated with purified human C1q (10 µg/mL). After incubation with increasing concentrations of purified DENV NS1 that was purified from the supernatant of BHK cells, bound NS1 was detected using a specific conformational monoclonal anti-NS1 antibody. (B) Microtiter plates were coated with purified human C1q (10 µg/mL). After incubation with increasing concentrations of purified DENV NS1 that was purified from *E. coli* cells, bound NS1 was detected with a specific polyclonal anti-NS1 antibody. Error bars indicate standard deviation from three independent experiments, and asterisks indicate significant difference from the control mock or BSA. *p<0.05, **p<0.01, ***p<0.001.

To assess whether NS1 directly binds C1q, an ELISA assay was designed in which the purified C1q was immobilized onto the plate and incubated with increasing concentrations of NS1 purified from the supernatant of DENV-infected BHK cells. As a control, we used the supernatant of mock-infected BHK cells subjected to an identical protocol used in the NS1 purification. To detect the interaction, we used a specific conformational anti-NS1 monoclonal antibody (DN1). We observed a significant increase in the optical density (OD) values when both proteins were incubated together, whereas no difference in the OD value was observed in the control experiment (Fig. 4A). Statistical analysis using two-way ANOVA revealed a significant difference between the control and binding curves. This result clearly indicates that NS1 binds C1q directly in a dose-dependent manner.

The NS1 protein is glycosylated in at least two sites [39] and coexists as different oligomeric states (dimers and/or hexamers). However, there is considerable discussion concerning which oligomeric state of NS1 interacts with other proteins and whether these interactions occur through its carbohydrate moiety. Previous studies from our group demonstrated that bacterial recombinant NS1 protein is able to form dimers in a conformation similar to those produced in insect cells depending on the refolding protocol used [34]. To evaluate whether recombinant NS1 purified from bacteria was able to bind C1q, we performed an identical experiment as described above for NS1 purified from BHK cells. As a control, we used purified bovine serum albumin (BSA). We observed that recombinant NS1 was also able to bind C1q directly in a dose-dependent manner similar to what was observed for NS1 purified from BHK cells. Note that the peak of the OD value measured for each NS1 protein was similar. We also performed an identical experiment but in the opposite order, i.e., immobilizing the NS1 protein (either from DENV-infected BHK or from recombinant bacterial cells) onto the plate followed by incubation with increasing concentrations of purified human C1q. These data were similar to those shown in Figure 4 (data not shown). Together, CoIP and ELISA results not only confirm the previous identification of C1q as an NS1-interacting partner but also reveal that this interaction occurs directly. Moreover, the finding that bacterial recombinant NS1 protein is also able to bind C1q indicates that this interaction is independent of posttranslational modification.

### Colocalization of the NS1 protein with endogenous C1q

**Figure 5:**
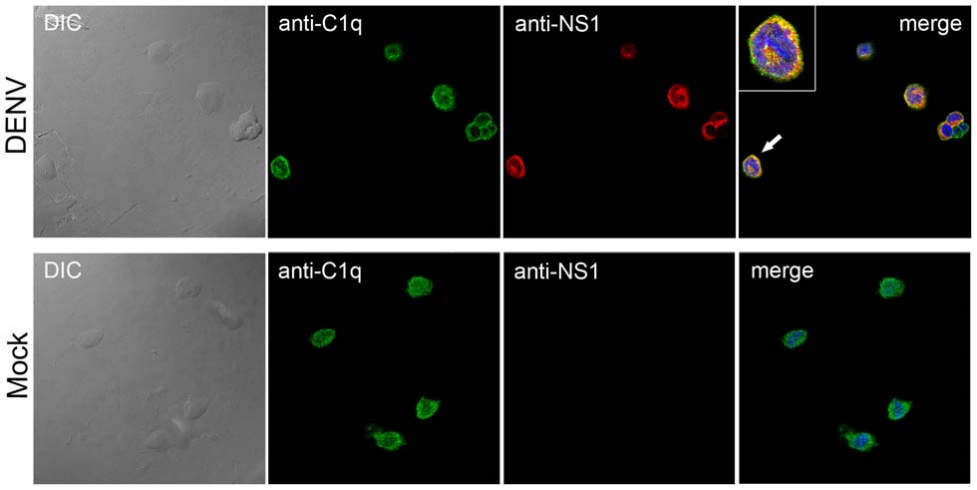
Colocalization of NS1 and C1q proteins by confocal microscopy. DENV2-infected THP-1 cells were labeled by incubation with polyclonal anti-NS1 (red stained) or monoclonal anti-C1q (green stained) antibodies. NS1 and C1q proteins were localized in vesicle-like structures in the cytoplasm, which is characteristic of secretory proteins. When the images were merged, distinct yellow regions were revealed, indicating colocalization of NS1 with C1q in these areas (detail). The subcellular localization of C1q was also analyzed in mock-infected cells, and it appeared at an identical position as that observed in DENV-infected cells, whereas no NS1 protein was detected in these cells. The cells were also incubated with DAPI for nuclear staining.

To evaluate whether these proteins colocalize during DENV infection *in vivo*, we performed an immunofluorescence assay of mock- and DENV-infected human acute monocytic leukemia (THP-1) cells using both anti-NS1 and anti-C1q antibodies. Although the liver is the principal production site of complement components, hepatocytes do not express the C1q protein [40]. In fact, it is the liver-resident macrophages (Kupffer cells) that are responsible for the production of C1q [41]. Alternatively, we used a mononuclear phagocytic cell line (THP-1) that has been shown to express C1q [42]. We observed that infected cells were labeled for both C1q (green) and NS1 (red) proteins (Fig. 5). When the images were merged, distinct yellow regions were observed, indicating the colocalization of NS1 with C1q in these areas (Fig. 5, detail). The C1q labeling was also observed in mock-infected cells, and it appeared at an identical position as that observed in DENV-infected cells in which no NS1 was detected. These results clearly indicate the colocalization of DENV NS1 with C1q in cell culture.

## Discussion

In the present study, we identified 50 putative interacting partners of DENV2 NS1 by screening a human liver cDNA library using a yeast two-hybrid system. In this screening, we detected the NS1 interaction with a key protein of the complement system, C1q. According to recent reports, the flavivirus NS1 seems to interact with complement system proteins and its regulators exhibiting immune-evasion functions, which contributes somehow to disease pathogenesis [32], [35], [36]. Based on this information, we focused our efforts on the confirmation of the DENV NS1 and C1q interaction by coimmunoprecipitation, ELISA and immunofluorescence assays. The complement system consists of more than 30 proteins that are present as either soluble blood-borne or membrane-associated proteins. This collection of proteins organizes into a hierarchy of proteolytic cascades beginning with the identification of pathogenic surfaces and leading to i) the generation of potent proinflammatory mediators (anaphylatoxins); ii) the opsonization (coating) of the pathogenic surface using various complement opsonins (e.g., C3b); and iii) targeted lysis of the pathogenic surface by assembling membrane-penetrating pores known as the membrane attack complex (MAC) [43], [44]. Viruses have developed strategies to limit or retard their recognition by the complement cascade and inhibit the immune response. These strategies include i) secretion of molecules that mimic or recruit host complement regulators and expression of surface proteins that interact with the antibody F_c_ region, thereby preventing C1q-dependent action; ii) incorporation of complement components on the virion; and iii) upregulation of complement regulatory proteins on the surface of infected cells [44]. It is known that C1q binds to the F_c_ region of the antibody on antigen-antibody complexes and activates the classical pathway in which C3 convertase promotes C3b-mediated opsonization and assembly of C5b-C9 MAC [37], [45], [46]. This pathway is also activated in the absence of antibodies due to the direct interaction of C1q with viral surface proteins, as observed for the p15E protein of oncornavirus [47] and gp41 and gp120 of the human immunodeficiency virus (HIV-1) [48], [49].

The flavivirus NS1 protein has been described as an immune evading protein [43] that binds complement regulators such as human clusterin, which inhibits MAC formation [50], and C4b-binding protein (C4BP), which regulates complement activation by interacting with C4b [36]. In addition, the NS1 protein binds complement proteins such as C4, also identified in this work, forming a complex with proC1s/C1s and leading to C4 degradation [32]. These interactions may help the virus to reduce complement activation via the classical and lectin pathways [32], [36]. Moreover, several studies have indicated that the complement system can restrict ADE. ADE is a phenomenon associated with dengue severity that occurs when non-neutralizing antiviral antibodies enhance viral entry into host cells, leading to increased efficiency of infection [4]. Experiments with mouse sera lacking individual complement components have indicated that C1q is sufficient to restrict ADE in West Nile virus (WNV) infection [51], and this has also been demonstrated using human sera in DENV infection [52]. C1q can also increase the potency of antibodies targeted against WNV by modulating the stoichiometric requirements for virus neutralization [53]. Our results indicated that DENV2 NS1 can directly bind the complement protein C1q, and this interaction is not dependent on the glycosylated form of DENV NS1. Nevertheless, further experiments are required to ascertain whether this interaction will modulate complement activation.

Interestingly, most of the identified NS1-interacting partners are proteins that are secreted to the extracellular milieu, such as plasminogen, haptoglobin, hemopexin, α-2-HS-glycoprotein, retinol binding protein 4, transferrin, and C4 and are classified as APPs. They actively participate in the acute-phase response (APR), and their plasma concentration varies during the inflammatory process. The APR is an important pathophysiological phenomenon that supersedes the normal homeostatic mechanisms during infection. The APR is an essential component of the innate immune system and plays an important role in limiting hepatic tissue injury. Several APPs can initiate, amplify or sustain inflammation, whereas others can attenuate it [54], [55]. Increasing the circulating levels of proinflammatory cytokines, chemokines, and immune effectors, for example, can lead to initiation of the inflammatory process. APR proteins crosstalk with proteins involved in the coagulation process, as observed during septic shock. This crosstalk is of great importance in the induction of episodes of DHF/DSS [56], [57]. Therefore, the NS1-plasminogen interaction would be a promising start point to study the abnormal activation of the fibrinolytic system associated with DHF/DSS events. There has been considerable discussion on the origin and maintenance of signals/effectors that trigger the vascular leakage phenomenon in DENV infection. However, the hypothesis that several factors, such as host genetic background, virus characteristics and immune response, contribute to dengue severity is commonly accepted [58]. During the course of DENV infection, increased levels of proinflammatory cytokines and chemokines are observed, including TNF-α, IL-1β, IL-6, IL-8, IL-10, IFN-γ, and more recently, the HMGB-1 [18] [10]–[17]. High levels of TNF-α and IL-6 influence disease severity and are directly correlated to the increase in vascular permeability [17]. It is known that the NS1 protein interacts with STAT-3β [59] and also activates the transcriptional regulator NF-κB [60]. These two proteins (NF-κB and STAT3) are likely to play important roles in the liver inflammatory response and in the maintenance of homeostasis, induction of APP synthesis by stimulating cytokines such as IL-6 and TNF-α, and in inducing liver dysfunction [54], [61]. However, further studies are required to elucidate the mechanism by which NS1 modulates APP and APR phenomena.

In summary, our results demonstrate that DENV NS1 directly interacts with C1q, a key protein of the complement system. The specific C1q-NS1 interaction may enable the virus to avoid complement activation through the classical pathway by possibly preventing antibody interaction and/or enabling C1-complex assembly. This hypothesis could be confirmed with *in vivo* studies by assessing complement activation in C1q, C1s or C1r-deficient mice challenged with DENV and/or inoculated with NS1 protein. Moreover, yeast two-hybrid screening revealed that DENV NS1 interacts with a wide range of proteins including some APPs that are important effectors of APR, which is an orchestrated mechanism focused on the reestablishment of normal liver and plasma homeostasis. Binding to and altering the properties of APPs may provide the molecular basis to link dengue infection and hemostatic abnormalities such as vascular leakage, thrombocytopenia and hemorrhage. However, further studies are necessary to confirm the role of NS1 interaction with C1q and APPs in the pathogenesis of DENV infection.

## Materials and Methods

### Cloning the ns1 gene into the pGBKT7 plasmid

The *ns1* gene, from the cDNA of the DENV2 New Guinea C (NGC) strain, was amplified by PCR using the forward primer 5′-AATAGGATCCATGATAGTGGTTGCGTTGTGAGC-3′ containing the *Bam*HI restriction site (underlined bases) and the reverse primer 5′-TATTGCGGCCGCTTAGGCTGTGACCAAGGAGTTGAC-3′ containing the *Not*I restriction site (underlined bases) and the TAA stop codon. The PCR conditions were as follows: 94°C for 2 min, 35 cycles of 94°C for 30 s, 58°C for 1 min and 68°C for 2 min followed by a final extension at 68°C for 7 min. The amplified gene was cloned in frame with the GAL4 DNA-binding domain (BD) of the yeast expression vector pGBKT7 (Clontech, CA, USA) to construct the bait plasmid (pGBKT7-NS1).

### Yeast two-hybrid screening

Using the full-length NS1 as bait, a yeast two-hybrid screening was performed against a human liver cDNA library fused to the GAL4 activation domain (AD) using the pACT2 vector and the Matchmaker GAL4 Two-Hybrid System 3 (Clontech, CA, USA). The yeast strain AH109 was transformed with the pGBKT7-NS1 plasmid using the lithium acetate method and then grown in SD (synthetic defined) medium lacking tryptophan (SD–Trp). Autoactivation of the *HIS3* reporter was confirmed by the growth of clones in SD medium lacking histidine, leucine and tryptophan (SD–His–Leu–Trp). The transformed cultures were then plated onto SD–His–Leu–Trp and SD–Ade–His–Leu–Trp media to select putative positives clones. The activity of the *lacZ* reporter gene was evaluated by the β-galactosidase assay (colony-lift filter) using the substrate X-gal on nitrocellulose membranes. To eliminate false positives, a plasmid linkage assay was also performed. The positive plasmids were sequenced, and their gene sequences were analyzed using the BLASTN and BLASTX software available at NCBI (www.ncbi.nlm.nih.gov).

### Cell culture and infection

Baby hamster kidney fibroblast (BHK; ATCC, USA) and human acute monocytic leukemia (THP-1; ATCC, USA) cell lines were cultured in α-MEM Medium (Gibco) and RPMI Medium 1640 (Gibco, NY, USA), respectively, supplemented with 10% fetal bovine serum (Invitrogen, NY, USA), 0.22% sodium bicarbonate and 0.2% HEPES, pH 7.4, in a humid CO_2_ incubation chamber at 37°C. After 2 days, BHK and THP-1 cells were mock-infected or infected with DENV2 strain 16681 at a multiplicity of infection of 2. All experiments were performed at 48 h post infection.

### Purified NS1

DENV2 NS1 was produced and isolated from the supernatant of BHK cells that were infected with the DENV2 strain 16881. The supernatant was harvested, and NS1 was purified as described previously [62]. DENV NS1 from *E. coli* was expressed and purified as described previously [34].

### Coimmunoprecipitation and Western blot

The coimmunoprecipitation was performed using a kit (Pierce, IL, USA). Purified anti-NS1 polyclonal antibody or IgG_1_ control antibody was attached to an N-terminal-binding resin. Purified NS1 from BHK supernatants and purified human C1q (Sigma-Aldrich, USA) were mixed and added to the anti-NS1-coated resin. C1q protein was also added to the anti-NS1- and IgG_1_-coated resin to serve as controls and to assess nonspecific binding. The samples obtained from the CoIP assay were separated by 12% SDS-PAGE and transferred onto a Hybond ECL nitrocellulose membrane (GE Healthcare, Sweden). The membrane was then blocked with 5% BSA in TBST (0.1% Tween 20 in TBS [25 mM Tris-HCl, pH 7.6, 3 mM KCl, and 140 mM NaCl]) for 2 h followed by overnight incubation with a mouse polyclonal anti-NS1 or mouse monoclonal anti-C1q antibody (Abcam, USA) in blocking solution. The membrane was then washed three times with TBST and incubated with anti-mouse IgG conjugated to horseradish peroxidase (Promega, USA) in blocking solution for 2 h. The membrane was washed again, developed with a Supersignal West Pico kit (Pierce, IL, USA) and exposed to Kodak MXG/PLUS film. The band intensity was measured by Scion Image software.

### DENV2 NS1 and C1q ligand binding ELISA

Purified human C1q (Sigma-Aldrich, USA) at a concentration of 10 µg/mL was adsorbed to wells in MaxiSorp microtiter plates (Nunc) at 4°C overnight. After five washes with phosphate-buffered saline (PBS), nonspecific binding sites were blocked with 200 µL of 1% BSA in PBS containing 0.05% Tween 20 (PBST) for 1.5 h at 37°C followed by five washes with PBS. Purified NS1 from BHK or *E. coli* at specific concentrations were added to each well and incubated for 2 h at 37°C. Plates were then washed five times with PBST followed by a 1-h incubation with a DENV2 NS1-specific monoclonal (Abcam) or polyclonal antibody (1∶1000 dilution). After five washes with PBST, anti-mouse IgG conjugated to horseradish peroxidase (Promega, USA) was added sequentially for 1 h at 37°C. After five final washes with PBST, the signal was developed by adding 100 µL of 0.4 mg/mL o-phenylenediamine (OPD) and 50 µL of 9 N H_2_SO_4_ stop solution to each well. The OD at 490 nm was determined by a 96-well plate reader.

### Statistical analysis

Datasets were compared by a two-tailed, unpaired Student *t* test and statistical significance was achieved when *p* values were <0.05. Multiple comparisons were performed using two-way ANOVA (Bonferroni post-test), and asterisks indicate significant difference from the control (*p<0.05, **p<0.01, ***p<0.001).

### Immunofluorescence confocal microscopy

THP-1 cells were washed in PBS, pH 7.2, and fixed for 30 min with 4% freshly prepared formaldehyde diluted in PBS. Cells were deposited on poly-L-lysine-treated microscope slides and permeabilized with 1.5% Triton X-100 in PBS, pH 7.2, for 25 min. The slides were incubated in blocking solution containing 1.5% BSA in PBS, pH 7.2, and then with purified anti-NS1 polyclonal and anti-C1q monoclonal antibodies diluted 1∶100 in blocking solution for 1 h. The cells were washed and incubated for 45 min with Alexa 488-conjugated goat anti-mouse IgG antibody (Invitrogen, USA) or with Alexa 546-conjugated goat anti-rabbit IgG antibody (Invitrogen, USA) diluted 1∶400 in blocking solution. The cells were subsequently incubated with 5 µM 4′,6-diamidino-2-phenylindole (DAPI, Sigma, USA) for 20 min at room temperature. The slides were mounted in N-propyl gallate and observed using a Leica TCS SP5 confocal microscope. All images were collected with LAS AF Lite 2.6 software (Leica Microsystems).
